# About Your Child’s Eating scale: a cross-cultural adaptation and validation of the questionnaire in the Greek language

**DOI:** 10.1017/jns.2024.83

**Published:** 2024-12-03

**Authors:** Andri Papaleontiou, Louiza Voniati, Alexandros Gryparis, Rafaella Georgiou, Vassiliki Siafaka, Dionysios Tafiadis

**Affiliations:** 1 Department of Speech & Language Therapy, School of Health Sciences, University of Ioannina, Ioannina, Greece; 2 Department of Health Sciences, Speech and Language Therapy, European University, Nicosia, Cyprus

**Keywords:** About Your Child’s Eating (AYCE), Feeding behaviours, Greek-Cypriot parents/caregivers, Parent/caregiver questionnaire, Paediatric eating and feeding process

## Abstract

Feeding is an interactive process between parents and children and is related to children’s healthy nutrition, growth, and feelings about the child or parent. The effectiveness of the interaction between feeding and behaviour is strongly influenced by how well this reciprocal procedure is stimulated and supported.

The current study aimed to cross-culturally adapt and validate the About Your Child’s Eating (AYCE) questionnaire in its Greek language version for Greek-Cypriot parents and caregivers of children aged six months to 16 years with or without feeding and swallowing problems.

One hundred Greek-Cypriot parents/caregivers of children with feeding and swallowing difficulties and 100 Greek-Cypriot parents/caregivers of children without feeding and swallowing difficulties participated in this study. This study was conducted at mainstream schools and private speech-language therapy clinics in Cyprus. According to WHO, the AYCE questionnaire was translated and culturally tailored for Greek speakers.

The analysis revealed a statistically significant difference between the AYCE total mean scores of parents in the Typical Development of Feeding Behaviors group (c-TDFB) (M = 44.03, SD = 11.18) and parents in the Atypical Development of Feeding Behaviors group (c-ADFB) (M = 63.56, SD: 16.22) (P < 0.001), with c-ADFB scoring significantly higher. The overall evaluation of the scale yielded a Cronbach’s α coefficient of 0.916.

The validity of the AYCE questionnaire in Cyprus was also assessed. The findings demonstrate that the AYCE can be a beneficial tool for determining critical facets of the feeding parent–child interaction for preschool- and school-aged Greek-Cypriot children in Cyprus.

## Introduction

Feeding is a relational and reciprocal process that hinges on the characteristics of both the parent/caregiver and child.^(1)^ It involves ongoing interaction and collaboration between the child and parent/caregiver.^(2)^

Early childhood and infancy require healthy feeding since this period is crucial for physical and neurological growth, while improper care might harm long-term development.^(3)^ Parents/caregivers have a high degree of control over their children’s environments and experiences throughout the feeding process. Their role is primarily to promote their children’s capacity for development and flourishing.^(4,5)^ Parents and caregivers are vital in shaping a child’s food intake pattern and eating manners over their intentional or unintentional feeding habits.^(6)^ It is well documented that healthy eating habits emerging in infancy and toddlerhood have a greater impact on family dynamics.^(7)^

Even if the collaboration of the parent/caregiver with the child is effective, the amount of nutritious intake consumed by a child with neurodevelopmental abnormalities may have further negative consequences on neurodevelopment.^(8)^ According to previous reports, approximately 20–30% of infants and toddlers tend to experience feeding-related problems, which increase the risk of nutritional imbalance and, thus, fail to grow.^(9)^

Feeding disorders can be caused by medical conditions and developmental deficits, with behavioural difficulties leading to additional implications.^(10)^ These disorders can manifest as disruptive, active, or passive behaviours that challenge familial relationships.^(11)^ Positive reinforcement can minimise these negative behaviours, but additional stress may exacerbate food rejection.^(12)^ Parents/caregivers must adapt their roles to detect these challenges and seek a speech-language pathologist’s (SLP) professional opinion for a multimodal assessment.^(12,13)^ These behaviours can include refusal to eat, intense crying, aversion to feeding methods, turning the head away, blocking the mouth, and even leaving the table.^(14,15)^

The role of the SLP is decisive in this multimodal assessment for determining the safest and most effective way for children to consume adequate dietary intake.^(16)^ Initially, information was collected via a thorough review of the developmental and feeding history.^(17)^ Additionally, the SLP will perform a clinical (bedside) swallowing examination (CSE)^(18)^ to assess oral skills and pharyngeal swallowing function. While CSE can screen for oral dysphagia symptoms and behaviours, an instrumental examination such as a videofluoroscopic swallowing study (VFSS) may be required.^(19)^

In addition to the significance of identifying feeding difficulties, along with the presence of other impacts, questionnaires can play an important role in screening because they allow for quick detection of the presence of dysphagia. According to the American Speech-Language-Hearing Association (ASHA), questionnaires can improve the assessment of children’s feeding challenges by providing extra evidence and assistance.^(20)^ Parent/caregiver questionnaires are the Pediatric Assessment Scale for Severe Feeding Problems (PASFSFP),^(21)^ which was designed to assess improvement in the development of oral feeding skills for children who need continued tube feeding, the Mealtime Behavior Questionnaire (MBQ)^(22)^ which evaluates the severity of certain behaviours at mealtime and About Your Child’s Eating (AYCE)^(23)^ which assesses positive, negative, and neutral parent/caregiver–child interactions at mealtime.^(24)^ Questionnaires can help identify challenging eating behaviours in children, such as food refusal, selectivity, and emotional responses, and detect those at risk of feeding difficulties.^(22)^ They can also identify physical, sensory, emotional, and psychological complications that can disrupt feeding practices.^(21)^

The AYCE provides information concerning three critical domains: environmental support and positive background, responses of the parents/caregivers, and the behavioural reaction of the child during the meal.^(23,25)^ The intricate connections between the child and the family system make such circumstances challenging to assess, thus explaining why this questionnaire was developed to identify the pattern of a child’s eating habits.^(23,25)^

Considering the above, this study aimed to cross-culturally adapt and validate the AYCE questionnaire in its Greek language version for Greek-Cypriot parents/caregivers of children aged six months to 16 years with or without feeding and swallowing problems. To determine which children were at an increased risk of eating difficulties during mealtime and to explore the associations between AYCE dimensions and child characteristics, such as age, gender, and medical diagnosis.

## Methods

### Participants

Two hundred parents/caregivers participated in the study which were further divided into two groups: (a) the parents/caregivers of children with Atypical Development of Feeding Behaviors group (c-ADFB, N = 100); thus, the clinical group, and (b) the parents/caregivers of children with Typical Development of Feeding Behaviors (c-TDFB, N = 100), the control group. Parents/caregivers of children between six months and 16 years of age and each participant provided personal data encompassing demographics (such as origin, family, and socioeconomic status).

The principals of public and special schools and directors of private speech therapy clinics were individually briefed, concerning the study’s procedures. Additionally, parents/caregivers were initially verbally informed about the study’s goals, confidentiality of the data, and their use solely for scientific purposes, and if they agreed to sign a consent form.

Moreover, medical information regarding the child’s feeding and swallowing difficulties was obtained. Their age, developmental history, diagnosis, and feeding habits have been previously documented.^(26)^ The SLP also evaluated the oral motor mechanism of each participating child and identified the type of feeding disorder (thus, oral sensory feeding disorder, oral motor feeding disorder, and oropharyngeal dysphagia),^(27)^ and whether this was triggered by a sensory disorder, neurodevelopmental, genetic, neurological problems, or psychomotor delay.^(27)^ In certain instances, a VFSS was advised.^(28)^

### Inclusion criteria

Parents/caregivers in the c-ADFB had to have a child aged 6 months to 16 years with feeding and swallowing difficulties, were native Greek-Cypriot speakers, demonstrated the ability to understand and complete the questionnaire, and were willing to return for a required post-test of the questionnaire, in ten days, to assess test–retest reliability.^(29)^ Regarding the possibility of being included, the c-TDFB had to meet all of the inclusion criteria, and his/her child did not exhibit any feeding and swallowing difficulties or had underlying medical, developmental, or genetic disorders.

### Measures

#### About Your Child’s Eating (AYCE)

The AYCE consists of 25-Likert scale items rated from 1 (never) to 5 (nearly every time), inquiring from the parents or caregivers about their beliefs and concerns regarding their child’s eating, the frequency of their child’s eating behaviours, their mealtime interactions with the child, and their feelings about mealtime. Items came from scenarios provided by dietitians and psychologists.^(23)^ It includes three dimensions: Child Resistance to Eating (CRE), a Positive Mealtime Environment (PME), and Parent/Caregiver Mealtime Aversion (PMA).

#### Montreal Children’s Hospital Feeding Scale

The Montreal Children’s Hospital Feeding Scale (MCH-FS is a 14-item screening questionnaire^(30)^ administered to detect probable feeding difficulties in children. It was initially standardised in English and later translated into other languages, confirming and establishing its validity and reliability.^(29,31–35)^ The current study used its pilot Greek form for external criterion validity.^(36)^

### Procedure

The research began with permission from the Licensed Content Publisher Elsevier to use the AYCE questionnaire for cross-cultural adaptation and translation in Greek. The Cyprus National Bioethics Committee (EEBK/EΠ/2019/95) and the University of Ioannina Bioethics Committee (Approval Number: 27829) approved and authorised the study.

The AYCE was then translated and culturally adapted following the published World Health Organization (WHO, 2020) guidelines.^(37)^

Following appropriate authorisation, the study was conducted in various settings, including public and special schools, university clinics, and private speech therapy clinics in Cyprus.

A pilot study was conducted to determine how straightforwardly the translated questionnaire could be used to enhance the general effectiveness and quality of the research. Subsequently, the translated questionnaire was pretested on 60 parents/caregivers of children aged between 3 and 9 years with (N = 30) and without (N = 30) feeding and swallowing issues.^(38)^

### Statistical analysis

The normality of the data distribution was tested using the Kolmogorov–Smirnov and Shapiro–Wilk tests. All normally distributed variables are expressed as mean (M) and standard deviation (SD), while non-normally distributed continuous data are reported using the median (25^th^_75^th^ percentiles). To compare the means of answers for AYCE for the two major groups, an independent sample t-test was used, while for the comparison of means between the study’s subgroups, a one-way ANOVA test was implemented.

The reliability analysis of the AYCE in Greek was estimated using (a) Cronbach’s α coefficient for the internal consistency of its dimensions and (b) Spearman’s rho correlation coefficient for its test–retest reliability. The external criterion validity of the AYCE total score and its three factors was evaluated using the MCH-FS questionnaire using Spearman’s rho coefficient. The validity of the AYCE in Greek was estimated using the Content Validity Index (CVI). The CVI was computed based on the answers of five SLPs specialising in paediatric dysphagia. The level of agreement between the expert SLPs for each item was divided by five (the total number of SLPs) to obtain the Item-CVI (I-CVI). Subsequently, the CVI Items were added and divided by 10 to obtain the total Scale-CVI (S-CVI). According to the literature, the range of 0,78 and above for I-CVIs and 0.8 for S-CVI was recommended to consider the scale’s excellent content validity.^(39)^ Moreover, to determine the cut-off scores of the AYCE questionnaire, a receiver operating characteristic (ROC) analysis was performed for the mean score of answers between parents/caregivers of children without feeding and swallowing disorders and parents/caregivers of children with feeding and swallowing disorders.

Principal component analysis (PCA) and confirmatory factor analysis (CFA) were used to represent a set of observed variables in terms of a smaller number of variables (factors). Specifically, PCA is often used as a dimensionality reduction method to reduce the dimensionality of an extensive data set into a smaller one that still contains most of the information in the large set. Following the specific analysis, we confirmed that the correlations among the 25 items had an absolute value less than 0.8; items with an absolute correlation value greater than 0.8 had to be omitted from the analysis. Kaiser–Meyer–Olkin (KMO) and Bartlett’s test of sphericity were used to assess the factorability of our data. Then, a scree plot was utilised to investigate the number of principal components to be used, after which PCA, was performed.

CFA borrows many of the same concepts from EFA; however, in this step, we pre-determine the factor structure and verify the structure initially revealed by PCA. Regarding model fitting, we used the robust maximum likelihood estimator for CFA. CFI, TLI (Tucker–Lewis Index), and RMSEA were employed as appropriate measures for the CFA model. Specifically, CFI is the Comparative Fit Index; values can range between 0 and 1 (values greater than 0.70 indicate a good fit). The TLI also ranges between 0 and 1, with values greater than 0.70 indicating a good fit. Finally, RMSEA is the root mean square error of approximation, and for RMSEA, a P-value > 0.05 shows a close-fitting model to the data.

All reported P-values were two-tailed, and statistical significance was set at P < 0.05. The analysis was conducted using IBM SPSS v.28.0 (IBM Corp. Released 2021. IBM SPSS Statistics for Windows, Version 28.0. Armonk, NY: IBM Corp) and RStudio 2023.03.1, and Build 446 (RStudio Team (2020). RStudio: Integrated Development for R. RStudio, PBC, Boston, MA URL http://www.rstudio.com/. We used the lavaan library in RStudio for CFA analysis.^(40)^

## Results

### Samples demographic data

A total of 200 parents/caregivers (162 mothers) participated in this study. There were 28 girls and 72 boys in the c-ADFB group, and 44 girls and 56 boys in the c-TDFB group. The ailments that caused feeding and swallowing problems for children in the c-ADFB included syndromes, acquired disorders, developmental disorders, and cerebral palsy (see Table 1).

**Table 1.** Medical diagnosis and feeding/swallowing disorder of the children, in the c-ADFB group

Medical group N (%)	Diagnosis
Acquired disorders N = 25 (25%)	Cerebellar atrophy (N = 5), Left-side lobotomy (N = 1), PKU (N = 1), hard of hearing (N = 1), severe delays in development (N = 17)
Developmental disorders N = 44 (44%)	ASD (N = 40), SLI (N = 2), neurodevelopmental disorder (N = 2)
Syndromes N = 16 (16%)	Fragile X syndrome (N = 2), Coffin–Siris syndrome (N = 1) DiGeorge syndrome(N = 1), Down syndrome (N = 5), Dandy–Walker syndrome (N = 1), Mowat–Wilson syndrome (N = 1), Rett syndrome (N = 2), 1q44 syndrome (N = 1), ArCapa syndrome (N = 1), medial agenesis (N = 1)
Cerebral Palsy ν = 15 (15%)	Cerebral palsy (N = 15)
Feeding/swallowing disorder, N (100%)	Oral sensory feeding disorder 41 (41%) Oral motor feeding disorder 43 (43%) Oropharyngeal dysphagia 16 (16%)

*Note*: ASD, autism spectrum disorder; PKU, phenylketonuria.

The groups in the current study had similar demographic characteristics data. Specifically, the median age of the children in the c-ADFB group was 7.60 (IQR: 4.25–10.925) and for the C-TDFB group was 6.85 (IQR: 4.20–9.40), P = 0.407. Likewise, the maternal median age of the c-ADFB group was 39.00 (IQR: 35.00–42.00), and that of the C-TDFB group was 37.00 (IQR: 35.00–41.50), P = 0.498. Similarly, the paternal median age of the c-ADFB group was 41.00 (IQR: 36.00–46.00), and for the C-TDFB group was 40.00 (IQR: 37.00–43.00), P = 0.980.

The chi-square test revealed non-statistically significant differences in the medical diagnosis (P = 0.932) and feeding/swallowing disorders of the children (P = 0.913) of parents/caregivers in the c-ADFB group in comparison with the parents/caregivers in the c-TDFB group.

### Comparison of means between subgroups

Statistically significant differences were found between parents/caregivers in the c-TDFB, and parents/caregivers in the c-ADFB, in the AYCE total score and in all its dimensions, with higher scores observed in the c-ADFB group. Specifically, a statistically significant difference was observed for the AYCE total mean scores between c-TDFB group (M = 44.03, SD = 11.18) and c-ADFB group (M = 63.56, SD: 16.22) (P < 0.001). Similarly, a statistically significant difference was observed for the AYCE dimension of CRE between the c-TDFB group (M = 21.29 SD = 6.34) and c-ADFB group (M = 30.44, SD: 8.64) (P < 0.001), for the AYCE PME dimension (c-TDFB: M = 7.43 SD = 2.81 and c-ADFB: M = 12.22 SD = 4.38) (P < 0.001) and for the AYCE PMA (c-TDFB: M = 7.11 SD = 2.59; c-ADFB: M = 11.68 SD = 4.35) (P < 0.001), respectively (see Table 2).

**Table 2.** Comparison of means between c-TDFB and c-ADFB for the AYCE total score and its three dimensions

	c-TDFB (N = 100)	c-ADFB (N = 100)		
	*M (SD)*	*M (SD)*	*t (198)*	P
CRE dimension	21.29 (6.34)	30.44 (8.64)	8.535	<0.001
PME dimension	7.43 (2.81)	12.22 (4.38)	9.917	<0.001
PMA dimension	7.11 (2.59)	11.68 (4.35)	8.465	<0.001
AYCE total score	44.03 (11.18)	63.56 (16.22)	9.912	<0.001

Abbreviations: M, mean; SD, standard deviation; CRE, Child Resistance to Eating; PME, Positive Mealtime Environment; PMA, Parent/Caregiver Mealtime Aversion; *P-value < 0.001.

One-way ANOVA variance was used for group effects according to the ***diagnosis of feeding problems*** in the study subgroups. The analysis revealed a significant main group effect (P < 0.001) in the AYCE *total mean scores* between the c-TDFB (M = 44.03, SD = 11.18), oral sensory feeding disorders (M = 66.24, SD = 16.67), oral motor feeding disorder (M = 62.27, SD = 17.23), and oropharyngeal dysphagia (M = 60.12, SD = 11.22) groups. The same significant main group effects (P < 0.001) were computed in the AYCE *CRE total mean scores* between the c-TDFB (M = 21.29, SD = 6.34), oral sensory feeding disorders (M = 31.48, SD = 16.67), oral motor feeding disorders (M = 30.11, SD = 9.27) and oropharyngeal dysphagia (M = 28.62, SD = 5.43); the AYCE *PME total mean scores* (P < 0.001) between the c-TDFB (M = 7.43, SD = 2.81), oral sensory feeding disorders (M = 13.19, SD = 4.59), oral motor feeding disorders (M = 11.53, SD = 4.30) and oropharyngeal dysphagia (M = 11.56, SD = 3.79), and the AYCE *PMA total mean scores* (P < 0.001) between the c-TDFB (M = 7.11, SD = 2.59), oral sensory feeding disorders (M = 12.12, SD = 5.15), oral motor feeding disorders (M = 11.37, SD = 4.75), and oropharyngeal dysphagia (M = 11.37, SD = 3.59)(see Table 3).

**Table 3.** Group effect of means between the study’s subgroups according to the diagnosis of feeding problems for the AYCE total score and its three dimensions

	c-TDFB (N = 100)	Oral sensory feeding disorders group (N = 41)	Oral motor feeding disorders group (N = 43)	Oropharyngeal dysphagia group (N = 16)		
	*M (SD)*	*M (SD)*	*M (SD)*	*M (SD)*	F (3,196)	P
CRE	21.29 (6.34)	31.48 (16.67)	30.11 (9.27)	28.62 (5.43)	24.849	<0.001
PME	7.43 (2.81)	13.19 (4.59)	11.53 (4.30)	11.56 (3.79)	30.260	<0.001
PMA	7.11 (2.59)	12.12 (5.15)	11.37 (4.75)	11.37 (3.59)	24.064	<0.001
AYCE Total Score	44.03 (11.18)	66.24 (16.67)	62.27 (17.23)	60.12 (11.22)	33.847	<0.001

Abbreviations: M, mean; SD, standard deviation; CRE, Child Resistance to Eating; PME, Positive Mealtime Environment; PMA, Parent/Caregiver Mealtime Aversion; *P-value < 0.001.

The one-way ANOVA variance was also used for group effects for the ***medical diagnosis subgroups***. The analysis revealed a significant main group effect in the AYCE *total mean scores* between the c-TDFB (M = 44.03, SD = 11.18), syndromes (M = 58.25, SD = 15.76), acquired disorders (M = 67.80, SD = 17.07), developmental disorders (M = 65.020, SD = 16.43), and cerebral palsy (M = 57.06, SD = 11.85) (P < 0.001). Subsequently, the one-way ANOVA analysis gave back a significant main group effect in the AYCE *CRE total mean scores* between the c-TDFB (M = 21.29, SD = 6.34), syndromes (M = 26.00 SD = 8.24), acquired disorders (M = 33.53, SD = 8.29), developmental disorders (M = 30.93, SD = 8.86), and cerebral palsy (M = 27.33, SD = 6.86), P < 0.001; the AYCE *PME total mean scores* between the c-TDFB (M = 7.43, SD = 2.81), syndromes (M = 11.5, SD = 4.06), acquired disorders (M = 13.00, SD = 4.78), developmental disorders (M = 12.68, SD = 4.53), and cerebral palsy (M = 11.06, SD = 2.76), P < 0.001, as well as for the AYCE *PMA total mean scores* between the c-TDFB (M = 7.11, SD = 2.59), syndromes (M = 10.31, SD = 4.31), acquired disorders (M = 13.26, SD = 4.87), developmental disorders (M = 11.70, SD = 4.88), and cerebral palsy (M = 10.60, SD = 3.92), P < 0.001 (see Table 4).

**Table 4.** Group effect of means between the study’s subgroups according to medical diagnosis for the AYCE total score and its three dimensions

	c-TDFB (N = 100)	Syndromes (N = 16)	Acquired disorders (N = 25)	Developmental disorders (N = 44)	Cerebral palsy (N = 15)		
	*M (SD)*	*M (SD)*	*M (SD)*	*M (SD)*	*M (SD)*	F (4,195)	P
CRE	21.29 (6.34)	26.00 (8.24)	33.53 (8.29)	30.93 (8.86)	27.33 (6.86)	21.144	<0.001
PME	7.43 (2.81)	11.5 (4.06)	13.00 (4.78)	12.68 (4.53)	11.06 (2.76)	26.181	<0.001
PMA	7.11 (2.59)	10.31 (4.31)	13.26 (4.87)	11.70 (4.88)	10.60 (3.92)	21.910	<0.001
AYCE total score	44.03 (11.18)	58.25 (15.76)	67.80 (17.07)	65.02 (16.43)	57.06 (11.85)	27.634	<0.001

Abbreviations: M, mean; SD, standard deviation; CRE, Child Resistance to Eating; PME, Positive Mealtime Environment; PMA, Parent/Caregiver Mealtime Aversion; *P-value < 0.001.

### Reliability, validity measures, and ROC analysis for the AYCE questionnaire

The internal consistency of the AYCE total score was estimated at Cronbach’s α = 0.916, indicating excellent internal consistency. The reliability measures, according to the Cronbach’s analysis of the AYCE, by item ranged from 0.907 to 0.932. Test–retest reliability was also computed using the Spearman’s *rho* correlation coefficient between the 1^st^ and second administration of the AYCE questionnaire. The analysis showed a strong correlation with the AYCE total score (r_s_ = 0.999, P < 0.001).

Additionally, the internal consistencies of the three dimensions of AYCE were estimated. The internal consistency of the CRE category was assessed with Cronbach’s α = 0.871, which is very good, and the reliability measures to the Cronbach’s analysis by item ranged from 0.841 to 0.929. This was further estimated consecutively for the PME and the Parent Aversion to Mealtime (PAM) categories with Cronbach’s α = 0.843 and α = 0.855. The reliability measures for PME ranged from 0.767 to 0.847 and PAM from 0.814 to 0.847.

To establish the content validity of the AYCE in the Greek Language, the S-CVI was computed and was equal to 1. The clearance of the questionnaire and the CVI for all items was one, and the total agreement was 25. The external criterion validity of the AYCE total mean score and its three factors was evaluated using the MCH-FS questionnaire using the Spearman’s r coefficient. The analysis returned statistically positive and robust results for the AYCE total mean score (r_s_ = 0.851, P < 0.001), AYCE *CRE* (r_s_ = 0.819, P < .001), AYCE *PME* (r_s_ = 0.729, P < 0.001), and AYCE *PMA mean scores* (r_s_ = 0.803, P < 0.001), respectively.

**Fig. 1.**
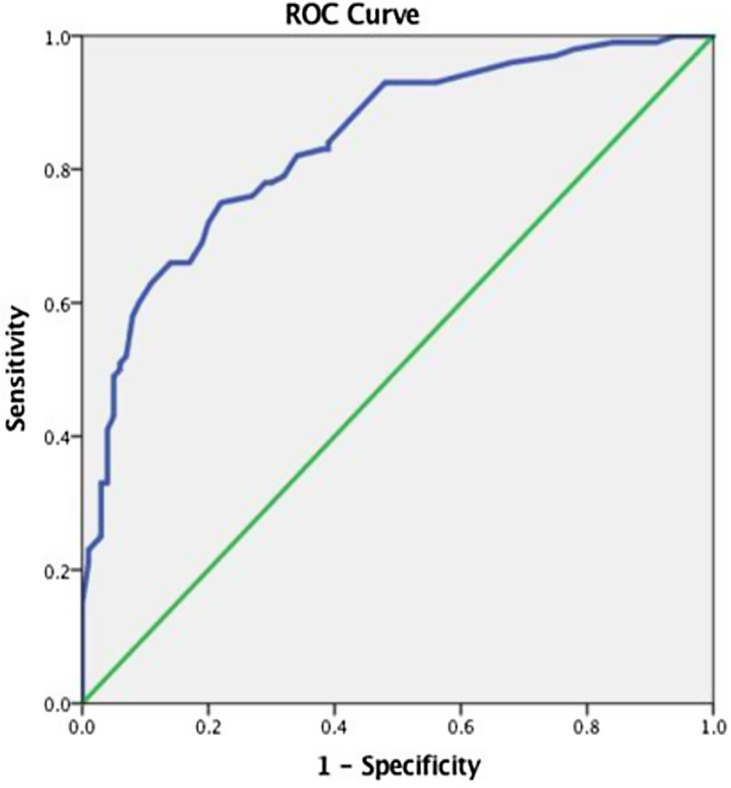
Receiver operating characteristics (ROC) curve for AYCE total score between the Control and the Atypical Development of Feeding Behaviors group (c-ADFB).

ROC analysis was further conducted to determine the cut-off points of the AYCE total score (see Fig. 1).

A statistically significant positive discrimination between the c-TDFB and the c-ADFB was revealed (*AUC* 0.839, (95% CI: 0.784–0.893), P < 0.001). The cut-off point was equal to 51.00 with a sensitivity of 0.750 and a 1-specificity of 0.220.

**Fig. 2.**
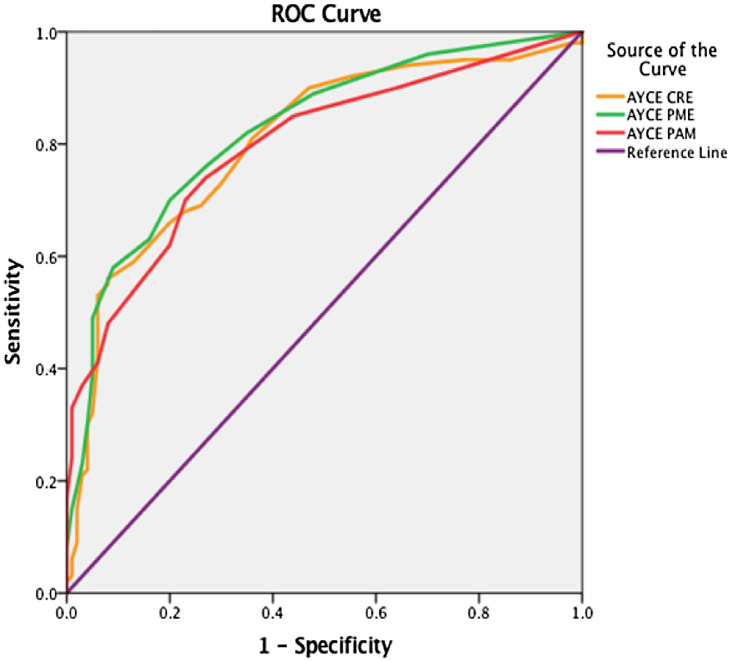
Receiver operating characteristics (ROC) curve for the AYCE CRE, the AYCE PME, and the AYCE PMA total mean scores, respectively.

The same analysis was conducted to determine the cut-off points of the AYCE *CRE,* AYCE *PME,* and AYCE *PMA total mean scores, respectively,* between the two main groups. A statistically significant positive discrimination was observed (*AUC* 0.804, (95% CI: 0.742–0.866), P < 0.001) and a cut-off point equal to 29.00 with a sensitivity of 0.560 and a 1-specificity of 0.008 for the AYCE *CRE total mean score*; for the AYCE *PME total mean score* (*AUC* 0.824, (95% CI: 0.767–0.882), P < 0.001) and a cut-off point equal to 10.00, with a sensitivity of 0.700 and a 1-specificity of 0.200; and the AYCE *PMA total mean score* (*AUC* 0.796, (95% CI: 0.735–0.858), P < 0.001) and a cut-off point equal to 9.00, a sensitivity of 0.700, and a 1-specificity of 0.230 (see Fig. 2).

### Principal component analysis (PCA) measures

We checked for all pairwise correlations between items to detect absolute correlations greater than 0.8. In our case, q4 was strongly correlated with q5. Hence, one had to be excluded from the rest of the analysis, and we decided to exclude q5.

The KMO method used to measure sampling adequacy is a better measure of factorability. According to Kaiser’s guidelines, the suggested cut-off for determining the factorability of the sample data is KMO ≥ 0.6. The total KMO in our data is 0.94, indicating that, based on this test, we can conduct a factor analysis. Next, we performed Bartlett’s Test of Sphericity estimated P-value < 0.001, which indicated that factor analysis may be helpful to our data. Additionally, the determinant of our data matrix was greater than zero, which implies that our factor analysis will probably run without any numerical issues.

Next, we investigated the scree plot to determine the number of components used in PCA. Considering the results from the scree plot, we decided to use three principal components because they explained most of the variability in the data. Table 5 presents the factor loadings for each of the 25 items on the three factors, along with h^2^, which is the proportion of each variable’s variance that can be explained by the principal components (e.g. the underlying latent continuum). By inspecting the items included in each factor, the three factors express the following variables: Factor 1, Child Resistance to Eating, factor 2, Positive Mealtime Environment, and Factor 3, Parent Aversion to Mealtime.

**Table 5.** Principal component analysis of the AYCE

Factor items	Mean	Rotated factor loading	h^2^
Factor 1 – Child Resistance to Eating			
1. My child hates eating	1.88	0.68*	0.63
2. I feel like a short-order cook because I have to make special meals for my child	2.17	0.63*	0.44
4. I feel that it is a struggle or fight to get my child to eat	2.13	0.71*	0.73
6. I worry that my child will not eat right unless closely supervised	2.33	0.59*	0.55
7. My child is a picky eater	3.13	0.71*	0.58
12. I dread mealtimes	2.22	0.65*	0.69
16. There are arguments between me and my child overeating	1.92	0.66*	0.58
17. My child seems to have no appetite	1.89	0.66*	0.59
19. My child refuses to eat a planned meal	2.18	0.77*	0.66
20. I have to force my child to eat	2.01	0.77*	0.72
21. I use preferred foods (such as dessert) as rewards or bribes to get my child to eat “good” foods.	1.85	0.53*	0.41
25. We end up grabbing meals whenever we can with no time for planning	2.13	0.54*	0.33
Factor 2 – Positive Mealtime Environment			
22. We watch television during meals	2.95	0.57*	0.43
23. There are house rules about how much kids have to eat (for example, the “Clean Plate Club”; No dessert until you eat what’s on your plate	2.29	0.52*	0.27
24. I have thought about putting my child on a diet.	1.43	0.69*	0.5
Factor 3 – Parent Aversion to Mealtime			
3. Mealtimes are among the most pleasant in the day	2.43	0.55*	0.52
8. The family looks forward to meals together	1.79	0.80*	0.73
9. My child enjoys eating	4.06	–0.58*	0.6
10. Mealtime is a pleasant, family time	1.94	0.78*	0.82
11. I get pleasure from watching my child eating well and enjoying his/her food	1.39	0.65*	0.46
13. We have nice conversations during meals	2.26	0.77*	0.61
15. It is hard for me to eat dinner with my child because of how he/she behaves	1.72	0.51*	0.49
18. My child has mealtime tantrums	1.73	0.53*	0.56

* Factor loadings above 0.40 are marked in bold, and the highest value is underlined (with the 25 items calculated in our PCA).

### Confirmatory factors analysis (CFA) measures

In the CFA, we used the PCA results. Namely, we investigated how well a CFA model fits the data in the three factors suggested by PCA. Specifically, based on the PCA results for each factor, we only retained the items that had absolute (loading) > 0.4; the rest of the items were excluded from the specific factors. For example, items q1, q2, q7, q16, etc. were excluded from pc3. Note that these items are used in other factors, such as pc1. Therefore, they are not completely excluded from the model.

**Fig. 3.**
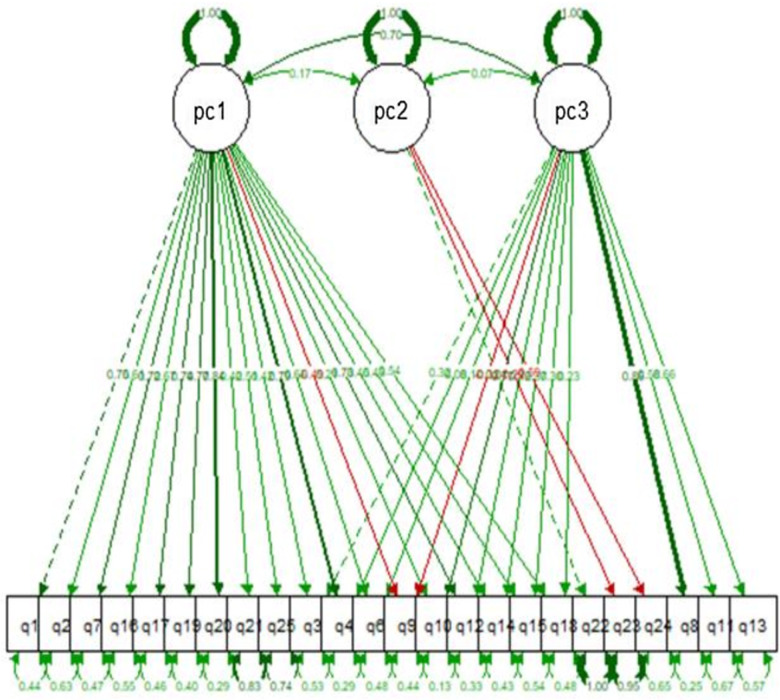
Visualisation of the path diagram of the model (showing the standardised coefficients).

Our CFA model fits without numerical issues. Figure 3 presents the path diagram of the estimated CFA model, while Table 6 shows the summary statistics for the rescaled (0–100) factors of interest. The summary statistics for this model are as follows: CFI = 0.931, TLI = 0.920, and RMSEA P-value = 0.061. These measures denote a very good fit between our data and the CFA model. Table 7 presents the Cronbach’s α results on each of the three factors and the 95% CI, all of which have a very good internal consistency (alpha > 0.843 in all cases). Table 8 presents the pairwise Spearman’s correlation coefficient between the three factors. As reported, all the coefficients are statistically significant.

**Table 6.** Summary statistics for the three factors and overall

Vars	M (SD)	Median	Min	Max
PC1	28.14 (24.47)	20.46	0	100
PC2	24.69 (24.53)	16.17	0	100
PC3	26.32 (24.36)	18.11	0	100
Overall	28.47 (25.65)	21.47	0	100

*Note*: PCA identified three factors: PC1 Principal Component Factor 1, PC2 Principal Component Factor 2, and PC3 Principal Component Factor 3.

**Table 7.** Cronbach’s α for the three factors and overall

Factors	Alpha	95% CI (bootstrapped based on 1000 samples)	
PC1	0.871	0.849	0.892
PC2	0.843	0.795	0.879
PC3	0.855	0.809	0.889
Overall	0.916	0.899	0.928

*Note*: PCA identified three factors: PC1 Principal Component Factor 1, PC2 Principal Component Factor 2, and PC3 Principal Component Factor 3.

**Table 8.** Spearman’s correlation coefficients results for the latent variables in the model

Factors	PC1	PC2
PC2	0.862^*^	
PC3	0.964^*^	0.917^*^

*Note*: PCA identified three factors: PC1 Principal Component Factor 1, PC2 Principal Component Factor 2, and PC3 Principal Component Factor 3.

* P-value < 0.05.

After rescaling the latent variables in the model to a 0–100 scale, the descriptive statistics for these variables are given in Table 6.

## Discussion

In the present study, we translated and adapted the AYCE questionnaire into Greek, a 25-item parent-report screening tool initially developed by Davies *et al.*.^(23)^ The AYCE assesses parents’ and caregivers’ beliefs and concerns about their children’s eating behaviours and family mealtime interactions. Crucially, it also serves as a detector of feeding disorders in children with both c-ADFB and c-TDFB.

We strictly adhered to the WHO’s translation and cross-cultural adaptation guidelines to ensure that our adapted version was suitable for Greek-speaking parents/caregivers, irrespective of their demographic, educational, or socioeconomic status. We strove to maintain semantic and conceptual equivalence with the original version, thereby preserving the content validity of the questionnaire.

### Validity and reliability of the AYCE-GR

The reliability test results for the AYCE-GR suggest that the tool was accurately translated into Greek. The reliability of the Greek translation closely aligns with that of the original English version, as indicated by the significant correlation coefficients, thus supporting our translation efforts.^(23)^ Our study also confirms that the AYCE-GR exhibits notably high internal consistency, echoing the findings from the original research and additional studies by Hendy *et al.*, 2018.^(41)^ Furthermore, our test–retest reliability analysis revealed robust temporal stability, which concurs with the findings of Hendy’s *et al.*.^(41)^ Utilising both PCA and CFA, our study reinforces the scale’s dimensionality and its capacity to effectively delineate the three distinct dimensions; CRE, PME, and PMA, along with an overall score.

### Internal consistency

Our psychometric examination of the AYCE-GR suggests excellent internal consistency, with a Cronbach’s α of 0.916. A detailed examination of the reliability measures by item showed alpha values ranging from 0.907 to 0.932. Each of the three dimensions of the AYCE-GR demonstrated very good internal consistency, with the CRE dimension showing a Cronbach’s α of 0.871, and the PME and PMA dimensions had alphas of 0.843 and 0.855, respectively. These findings align closely with those reported in the original study by Davies *et al.*, 2018.^(41)^ They are also, supported by robust internal and test–retest reliability results for the AYCE subscales in Hendy’s study.^(41)^ The construct validity of the AYCE-GR, as affirmed by the CVI analysis, underlines the robustness and appropriateness of the adaptations made for the Greek context.

### Group comparisons

Utilising the refined AYCE-GR measures, further statistical analysis revealed significant differences between the c-TDFB and c-ADFB groups across all measured dimensions. Specifically, the total mean scores for the c-ADFB group were consistently higher than those for the c-TDFB group, indicating more severe feeding issues in the clinical group.^(42)^ Using a one-way ANOVA, comparisons of the mean scores across the various medical diagnosis subgroups demonstrated significant main effects for all measures. These results suggest that AYCE-GR effectively differentiates between groups based on the severity of feeding disorders, which is a crucial capability for clinical assessment.^(43)^

### Limitations

A study’s reliance on parent/caregiver-reported data may introduce a degree of bias, potentially affecting the overall validity of the findings. Aside from the possibility of under-reporting food intake or eating issues, many parents/caregivers may not have been fully aware of the correct intake levels, which could have influenced the results. Additionally, the limited sample size and small medical diagnosis subgroups may confine the generalisability of the findings to a broader population.

### Future directions and clinical implications

Future research is needed to support the utility of AYCE-GR across a wider range of clinical samples and age groups. Replicating our findings in different clinical settings and comparing AYCE-GR factor scores to actual observed mealtime interactions would enhance the applicability and reliability of the tool. Expanding the administration of the questionnaire to various groups will also improve the validity and dependability of the research findings, providing essential insights for clinicians and researchers to manage feeding disorders more effectively.

## Conclusion

The results showed that the AYCE-GR is a reliable parent/caregiver completed questionnaire, suitable for children diagnosed with various medical disorders commonly presenting with c-ADFB.

Clinicians can easily administer the questionnaire while achieving the objectives described in the original scale and having appropriate psychometric measures to identify feeding disorders in Greek-Cypriot children from 6 months to 16 years of age. The positive cultural and psychometric testing results of the AYCE-GR version increase the scale’s eligibility for implementation in clinical and school settings. Further research is needed to support the utility of AYCE-GR. Replicating our findings in clinical samples of children with feeding disorders and other age groups would be helpful, as would comparing AYCE-GR factor scores with actual observed mealtime interactions.

## Data Availability

The data underlying this article cannot be shared publicly because of the privacy and confidentiality of the study participants, which are protected. The data of this study are available upon reasonable request from the corresponding author and the signing of a data transfer agreement.

## Permission to reproduce material from other sources

Elsevier, the Licensed Content Publisher, has granted us the copyright to use the AYCE questionnaire and has offered us permission to validate and culturally adapt it in Greek language and culture.
